# Myasthenia Gravis with Elderly Onset at Advanced Age

**DOI:** 10.7759/cureus.6808

**Published:** 2020-01-29

**Authors:** Otto Jesus Hernandez Fustes, Carlos Arteaga Rodriguez, Olga Judith Hernandez Fustes

**Affiliations:** 1 Neurology, Complexo Hospital de Clínicas - Universidade Federal do Paraná (CHC- UFPR), Curitiba, BRA; 2 Neurology, Universidade Positivo, Curitiba, BRA; 3 Neurophysiology, Clinica Neurológica das Américas, Curitiba, BRA

**Keywords:** myasthenia gravis, elderly onset, electroneuromyography

## Abstract

Myasthenia gravis (MG) in older adults has not been extensively studied. The prevalence of this disease in older people seems to be higher in recent epidemiological studies. In patients with disease onset after the age of 70, the diagnosis is more difficult as other conditions are more easily taken to be the causal element. The mortality is higher than in young patients, so prompt specific treatment can improve prognosis. We present an 85-year-old female patient with speech disturbance and difficulty in swallowing solids, and neurological examination with palpebral ptosis, disphonia, convergent strabismus and weakness and mild progressive fatigue in arms, with electroneuromyography and acetylcholine receptor antibody who was diagnosed with MG, emphasizing the importance of this entity in geriatric patients.

## Introduction

According to the National Household Sample Survey 2015, the number of elderly in Brazil reached 29,374,000 people, equivalent to 14.3% of the general population, Paraná is the state with the ninth largest elderly population in the country, consisting of 1,637,000 individuals, representing 14.6% of the general population [1]. As the aging population increases, health services must prepare for this new reality.

Myasthenia gravis (MG) forms the largest disease group of neuromuscular junction disorders and is caused by pathogenic autoantibodies to components of the postsynaptic muscle endplate [2-3] characterized by fluctuating muscle weakness and abnormal fatigability. Its prevalence is 7-20 cases per 100,000 and its incidence is 0.5 cases per 100,000 [4-5].

Myasthenia gravis is encountered at all ages but classically the distribution is bimodal: the first frequency peak between 20 and 40 years old at predominantly female dominance; the second frequency peak between 55 and 75 years old at male dominance [6-7].

Late presentation forms, above 75 years of age, have been reported. In the geriatric age group, the diagnosis is often delayed by the scarcity of information in the literature, difficulty of recognizing the typical symptoms and signs owing to the physiologic changes that occur with aging and by the large number of entities that are first thought for differential diagnosis. The problem of underdiagnosis is believed to be of greater importance in patients older than 80 years [8-9]. A survey in a local area of Japan clearly showed that late-onset MG has been increasing and a nationwide epidemiological survey in Japan also revealed that the ratio of late-onset MG (onset after 50 years) had increased from 20% in 1987 to 42% in 2006. However, the clinical and immunological differences between early and late-onset MG in Japan have not been elucidated [10]. We present a case of MG of elderly onset at advanced age.

## Case presentation

A 85-year-old woman from Curitiba, Brazil, who had no previous illness, was admitted to the ED of the hospital due to dysphonia and dysphagia to swallow solids, which started 20 days ago. Neurological examination showed bilateral eyelid ptosis, convergent strabismus due to paralysis of the rectus lateralis muscle, and slight decrease in proximal muscle strength in the upper limbs (Grade 4 MRC), which was accentuated by the repetition of the movements. Laboratory tests (blood count, erythrocyte sedimentation rate, rheumatoid factor, antinuclear factor, muscle enzymes, liver function tests, and thyroid) were normal (Table 1).

**Table 1:** Demographic, clinical, and laboratory test characteristics. n/a= no pathological alteration; MG=myasthenia gravis; MGFA=Myasthenia Gravis Foundation of America

Clinical case	
Age, yrs	85
Gender	F
Age at onset of MG, yrs	85
Anti-AchR-ab nmol/L	4.59
MGFA-score	II-A
Chest Rx	n/a
Chest CT	n/a
Brain MRI	n/a
Liver function tests	n/a
Thyroid function tests	n/a
Creatine phosphokinase	41 U/L
Rheumatoid factor	Non Reagent

**Figure 1:**
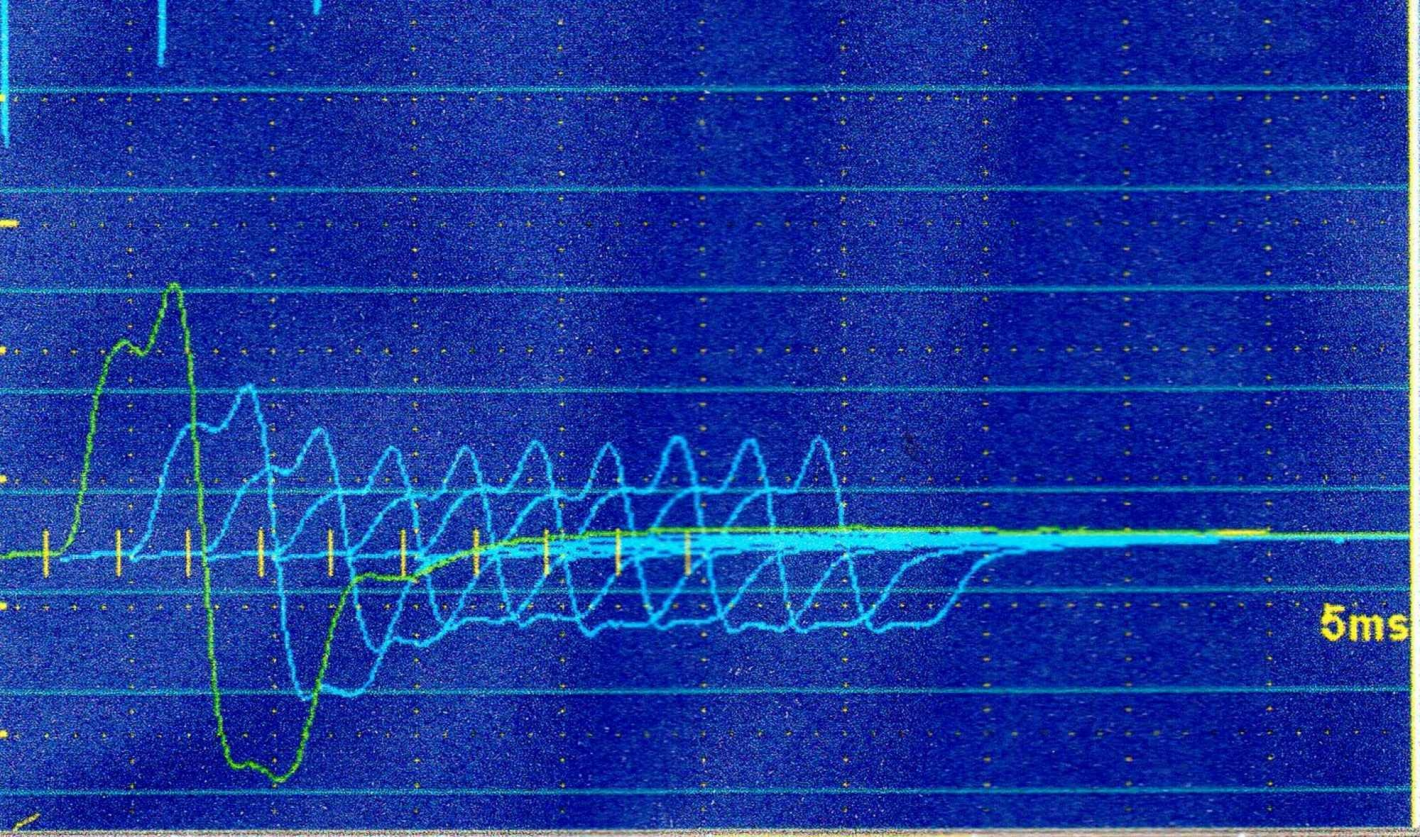
Repetitive nerve stimulation studies show an abnormal decrement.

**Figure 2:**
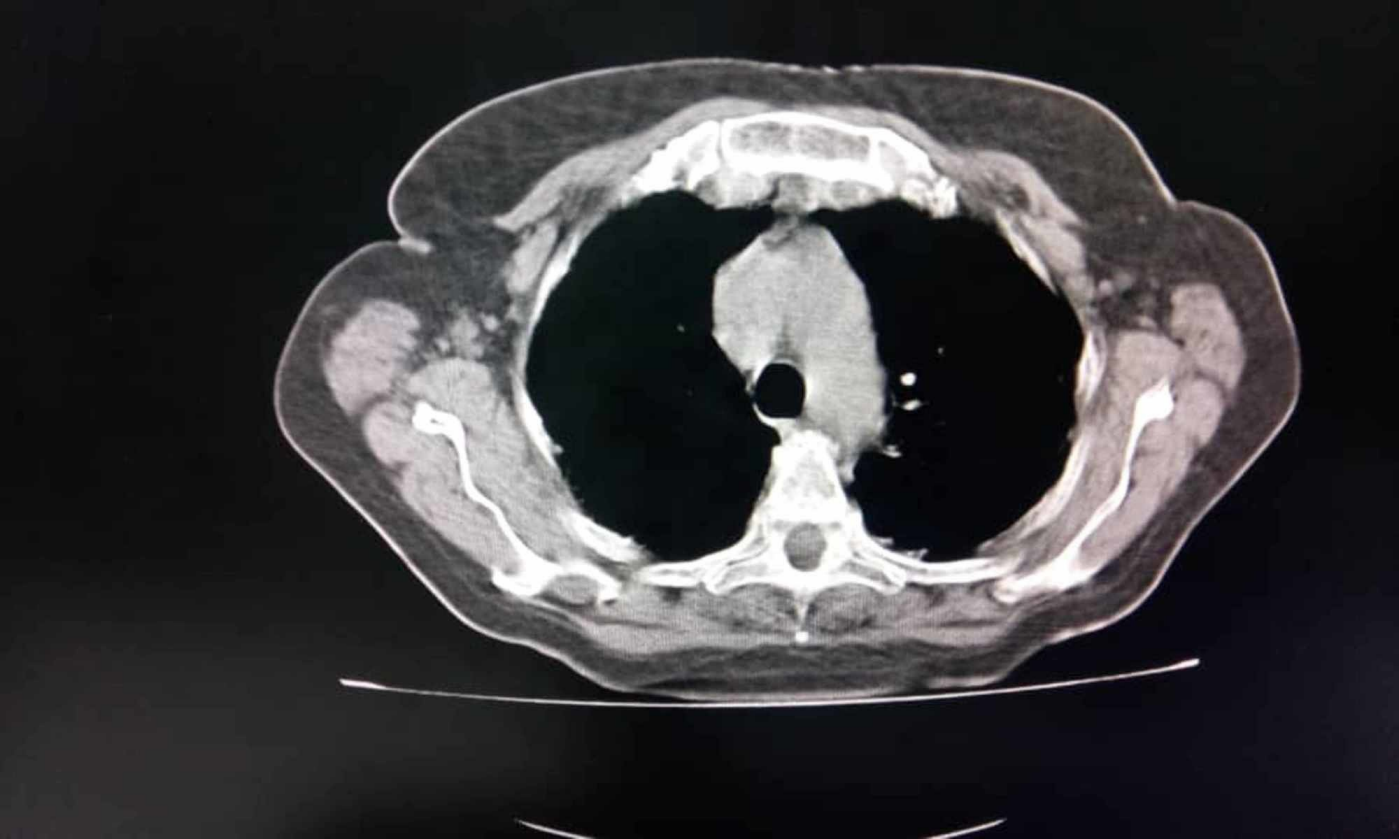
Chest CT.

Cranial magnetic resonance showed no changes. Electroneuromyography showed signs of distal sensory-motor polyneuropathy in the lower limbs without signs of denervation. The repetitive stimulation test showed a decremental pattern in the facial and spinal accessory nerves (Figure 1). Acetylcholine receptor antibody was positive, 4.59 nmol/L (reference value up to 0.8 nmol/L). To rule out the presence of thymoma, a chest Rx and CT scan (Figure 2) were performed which showed no expansive lesions in the mediastinal compartments. With a diagnosis of mild generalized MG (IIA), treatment with pyridostigmine was started with clinical improvement.

## Discussion

The prevalence of MG in older people appears to be higher in recent epidemiological studies than in previous reports. This finding may reflect the increasing age of the population, better diagnosis, and longer patient survival. However, a genuine increase in incidence in the elderly cannot be excluded [4, 8-9]. The high prevalence of previously unrecognized positive AChR-Ab in those older than 75 years suggests that MG might be substantially underdiagnosed in the elderly or that the incidence of late-onset MG in general may be increasing, or possibly both [11].

About 90% of the cases present with eyelid ptosis and diplopia, and ocular symptoms are the first to manifest in more than 50% of patients [7, 12-13] as reported in our patient, which was classified in Group 2a: generalized mild, according to Osserman and Genkins [14]. Patients with MG should be classified into subgroups, with implications for diagnosis, optimum therapy, and prognosis [15].

In our country there is no previous case study in patients with very elderly onset MG. A retrospective chart review in Ceará State, Brazil, from October 1981 to June 2009, shows that 122 patients were studied, and age at the first symptoms varied from 0 to 74 years (31.9 ± 14.4 years) [16].

As the elderly patient usually has comorbidities, and MG being a rare disease, differential diagnosis is important. Conditions that cause somatic muscle weakness should be considered, including congenital myasthenic syndromes, drug-induced myasthenia, hyperthyroidism, Graves' disease, Lambert-Eaton myasthenic syndrome, botulism, progressive external ophthalmoplegia, and intracranial expansive lesions [17]. Cerebrovascular disease, vertebrobasilar insufficiency, cervical arthrosis myelopathy, amyotrophic lateral sclerosis (bulbar form), Parkinson's disease, and generalized fatigability of depression should also be excluded.

In the past, MG was considered a disease unresponsive to therapy and associated with high mortality rates. This situation began to change in 1934, when physostigmine was used by Mary Broadfoot Walker, as an anticholinesterase agent for MG treatment. Thus, pyridostigmine has become the drug of choice in the treatment of MG since 1954. Currently, pyridostigmine has been cited as associated with the therapy in MG patients in more than 8000 articles in Google Scholar database [18].

Treatment in elderly patients has been preferentially drug-induced, with immunosuppression most of the time, due to the severity of the disease presentation and attention should be paid to the side effects of these drugs in the geriatric age group [19].

For treatment of elderly patients, azathioprine appears to be effective and better tolerated, while adverse effects of steroids are particularly frequent and severe at this age. Plasmapheresis has been reserved for severe acute cases [20]. Most patients with MG do well and have well-controlled disease. However, most need long-term and often life-long drug treatment with acetylcholinesterase inhibitors and usually low-dose immunosuppression. The high number of factors associated with muscle function in MG should drive future research towards an individually adapted treatment approach based on biomarker (autoantibody) assessment and monitoring [11].

## Conclusions

Our study shows a patient who started at 85 years of age with seropositive MG, not associated with thymoma, who responded satisfactorily to the use of pyridostigmine. The difficulty of establishing the diagnosis of MG is linked to the multiplicity of differential diagnoses encountered in the geriatric population. However, the practice simple complementary exams (electromyogram with repetitive nerve stimulation studies and AChR-Ab) allow to establish the diagnosis and to have use of symptomatic treatment, in general well tolerated and satisfactorily effective. The late onset forms of MG in the elderly over 80 years of age has drawn special attention with the aging of the population, and the neurologist and medical team should be aware of this possibility, as early diagnosis and treatment may change the prognosis in this age group.
